# Incidence, Knowledge, Attitude and Practice Toward Needle Stick Injury Among Health Care Workers in Abha City, Saudi Arabia

**DOI:** 10.3389/fpubh.2022.771190

**Published:** 2022-02-14

**Authors:** Abdullah Alsabaani, Norah Saeed S. Alqahtani, Sarah Saeed S. Alqahtani, Jawaher Hussain J. Al-Lugbi, Malak Ali Saleh Asiri, Shyamaa Elsayed Elaraby Salem, Ali Ahmed Alasmari, Syed Esam Mahmood, Mesheil Alalyani

**Affiliations:** ^1^Department of Family and Community Medicine, College of Medicine, King Khalid University, Abha, Saudi Arabia; ^2^Oral Surgery Department, New York University, New York, NY, United States; ^3^General Surgery Department, Armed Forces Hospital, Khamis Mushait, Saudi Arabia; ^4^Obstetrics & Gynaecology Department, Armed Forces Hospital, Khamis Mushait, Saudi Arabia; ^5^Pediatric Neurology Department, Abha Maternity and Pediatric Hospital, Abha, Saudi Arabia; ^6^Occupational Health Clinic, Infection Control Department, Aseer Central Hospital, Abha, Saudi Arabia; ^7^The Residency Program for Saudi Board in Community Medicine, Abha, Saudi Arabia; ^8^College of Nursing, King Khalid University, Khamis Mushait, Saudi Arabia

**Keywords:** incidence, needle stick injury, health care workers, Abha, attitude, practice, bloodborne diseases, safe injection

## Abstract

**Background:**

A needle stick injury is a serious occupational health hazard in health care settings. Health care workers are at risk of bloodborne diseases and the psychological consequences of these injuries. This study aims to estimate the incidence of needle stick injuries among healthcare workers during the previous 12 months and to assess their knowledge, attitude, and practice toward these injuries.

**Methods:**

This cross-sectional study was conducted from 1st August 2019 till 15th February 2020, and included 786 healthcare workers in Abha city, Saudi Arabia. A structured questionnaire was used to collect the data.

**Results:**

The incidence of needle stick injury among healthcare workers during the previous 12 months was (91/786) 11.57%. Nurses, females, and Saudis reported most needle stick injuries. More than half (52.7%) of the injuries went unreported. About 52.7% of needle stick injuries occurred during using sharp devices, and 42.9% of injuries happened in the patient room. The incidence of needle stick injury was significantly higher among those working at the secondary healthcare level (*p* = 0.003) and those practicing surgery (*p* < 0.001). Out of 786 participants, 94.7% knew the definition of needle stick injury, and 81.0% were aware of the procedure and guidelines to follow on sustaining a needle stick injury. Only 61.2% recognized that the recap of the needle is not recommended. Almost half of the participants (47.1%) agreed, and 33.6% strongly agreed that needle stick injury is preventable. A majority of healthcare workers (89.1%) had been vaccinated against Hepatitis B. Nearly 27.5% of healthcare workers incorrectly practiced recapping the needles with two hands and 8.7% bent needles before disposal. Recapping the needles was statistically significantly higher among healthcare workers who had a history of needle stick injury (*p* = 0. 046).

**Conclusion:**

Needle stick injury and its under reporting among healthcare professionals is still a prevalent risk. Raising awareness among healthcare workers and improving the reporting systems for needle stick injuries to ensure more protection and early use of post-exposure prophylaxis is required. Implementation of safety precautions and safe injection practices and providing engineered safety devices may further reduce the risk.

## Background

A needle stick injury (NSI) is a penetrating or cut wound in the skin caused by a needle or sharp instrument in the health care setting. Health care workers (HCWs) are at risk of accidental NSIs and sharp injuries because of the nature of their work. NSI is a severe occupational health hazard worldwide and around 3 million HCWs sustain NSIs and/or sharps injuries each year (1). In the USA, up to 800,000 sharp injuries have been estimated each year (2). In 2011, US EPINet™ reported 16.5 injuries per 100 occupied beds in 23 hospitals (3).

The rate of NSIs in the Kingdom of Saudi Arabia (KSA) at the national level was reported to be 3.2 per 100 occupied hospital beds in a study conducted during 2012 involving 52 hospitals (3). Analysis of reported data from King Saud Medical City in the Riyadh region shows a high rate of 13.8 NSIs per 100 occupied hospital beds during 2009 (4). Different rates have been reported from various health care institutions in other regions in KSA based on recorded data for reported injuries (5–10). However, these rates may underestimate the actual situation because injuries may usually go unreported. A review of studies on injury rates in the United Kingdom shows the difference between estimated rates and what was reported was up to 10-fold (11).

These injuries are a major source of infections with blood-borne diseases like Hepatitis B Virus (HBV), Hepatitis C Virus (HCV), and Human Immunodeficiency Virus (HIV) (12). The risk of transmission of this infection after exposure to percutaneous injuries with infected blood is 2–40% for HBV, 2.7–10% for HCV, and 0.3% for HIV (13). Additionally, studies show an influence on the mental health of the injured HCWs. Anxiety, depression, and worry about being infected or transmitting the infection to their family affected their quality of life (14, 15).

Health care institutions must take preventive measures to reduce this risk among HCWs. Education to raise awareness among health workers, training them on universal safety precautions, safe injection practices, sharp waste disposal, and provision of engineered safety devices have been reported to reduce such incidents by 62% in a meta-analysis study (16). In addition, according to UK guidelines, sound reporting systems for injuries and early use of post-exposure prophylaxis will reduce the risk of HIV infection (17).

Studies have been conducted to assess the incidence and prevalence of NSIs among health workers in KSA. However, these studies either have been among specific workers or are limited to localized institutions (18–23).

Therefore, up to our knowledge, no previous study has been conducted to evaluate the incidence/prevalence of NSIs among health care workers from different specialties at different levels of health care in KSA. This study was undertaken to estimate the incidence of NSIs among HCWs of different specialties at primary, secondary, and tertiary healthcare levels in Abha city, KSA. The knowledge, attitude, and practice of these HCWs toward NSIs and sharp object injuries were assessed.

## Materials and Methods

### Study Design and Setting

This analytical cross-sectional study was conducted from 1st August 2019 to 15th February 2020 among HCWs, who currently work in Abha city, Aseer Region, KSA. Different healthcare institutions from different healthcare levels (primary, secondary, and tertiary healthcare levels) were included.

### Inclusion and Exclusion Criteria

Different professions like physicians and nurses were included. HCWs grades such as consultants, specialists, or residents were encompassed. However, interns, medical and health-college students were excluded.

### Sample Size

A total sample of 786 HCWs (231 physicians including dentists, and 555 nurses) was required to estimate the expected rate of 50% NSIs among HCWs (24). A margin of error of 5% at a 95% confidence level and a design effect of 2 was considered for calculating the sample size. The sample units were selected from the different health facilities and PHCCs using a stratified approach based on sample probability proportionate to the size method.

### Sampling Technique

A stratified multistage cluster sampling technique was applied. The first stage of stratification was at the levels of healthcare institutions [primary (12), secondary (2), and tertiary (1) healthcare institutions]. The second stage was according to the profession of HCWs (physicians and nurses). A simple random sampling technique was used to select HCWs from each stratum.

### Sampling Frame

Sampling frame Fifteen healthcare institutions were included (12 primary, 2 secondary, and 1 tertiary healthcare institutions) with a totalsampling frame of 2,205 physicians and nurses. Of whom Fifteen healthcare institutions were included (12 primary, 2 secondary, and 1 tertiary healthcare institutions) with a total sampling frame of 2,205 physicians and nurses (Table 1).

**Table 1:** The sample size and sampling frame.

**Healthcare institutions**	**Total physicians**	**Total nurses**	**Total healthcare workers**	**Total sample**	**Physician sample**	**Nurse sample**
Tertiary healthcare hospital	355	743	1,098	335	98	237
Secondary healthcare hospitals	192	526	718	289	94	195
Primary Healthcare Centers	103	286	389	162	39	123
Total	650	1,555	**2,205**	**786**	**231**	**555**

### Data Collection Tools

Data were collected by using a structured questionnaire that was developed by the investigators. The questions were derived from The Saudi Ministry of Health guidelines (25, 26). Questionnaires were tested for their clarity, feasibility, and practicability. Four academic experts from King Khalid University, Abha, assessed content validity, and some minor modifications were made.

The final tested questionnaire consists of 39 questions with five components. The first part obtained information about HCW's socio-demographic data such as age, gender, nationality, and years of work practice. In the second part, 12 questions regarding HCWs experiences toward NSIs and the circumstances relating to the injuries, such as type of device, time, place, of injuries were assessed. In the third part, the knowledge of HCWs toward NSI was assessed based on their responses to questions related to the prevention and risk factors, disease transmission, and post-exposure measures. In the fourth part, HCWs attitudes toward NSIs were assessed based on their responses to statements using the Five-point Likert scale approach.

In the last part, HCW's practice toward NSIs was assessed based on their responses to 6 closed-ended questions with “yes” or “no” responses.

### Data Analysis

Statistical Package for Social Sciences (SPSS) version 25.0 was used for data entry and analysis. The data were described as frequencies and percentages for categorical variables. A chi-square test or Fisher test was used to test for associations between categorical variables, and Mann–Whitney U test was used to test for associations between ordinal variables. *P*-values < 0.05 are considered statistically significant.

### Ethical Considerations

All necessary official permission and ethical approval were obtained. The study's objectives were explained to all participants and assured them their responses would be fully confidential. A written informed consent form was obtained from each participant before administering the study questionnaire. Research teams distributed and collected the questionnaire manually on the same day.

## Results

Seven hundred and eighty six HCWs from different levels of health care completed the survey questionnaires. Out of the total, 62% were Saudis, 71% were females and 70.6% were nurses. About 44.7% of them were within the age range from 30 to 39 years. Regarding years of practice, 31.8% had between six to 10 years of experience, and 19.3% had less than two. About 81.3% were residents or general practitioners in terms of position, while specialists and consultants were 11.3 and 7.4% respectively. A higher proportion (42.6%) of HCWs were from a tertiary healthcare hospital (Table 2).

**Table 2:** Socio-demographic characteristics among the HCWs.

**Variables**	**Total HCWs**	**HCWs with NSI**	*p* value^*^
	***N* = 786**	***N* = 91**	
Gender			>0.05
Male	226 (28.8)	28/226 (12.4)	
Female	560 (71.2)	63/560 (11.3)	
Nationality			>0.05
Saudi	487 (62.0)	57/487 (11.7)	
Non-Saudi	299 (38.0)	34/299 (11.4)	
Profession			>0.05
Physician	231 (29.3)	28/231 (12.1)	
Nurse	555 (70.6)	63/555 (11.4)	
Position			>0.05
Consultant	58 (7.4)	4/58 (6.9)	
Specialists	89 (11.3)	11/89 (12.4)	
Resident/general	639 (81.3)	76/639 (11.9)	
Age			>0.05
20–29	282 (35.9)	37/282 (13.1)	
30–39	351 (44.7)	43/351 (12.3)	
40–49	96 (12.2)	7/96 (7.3)	
≥50	55 (7.0)	3/55 (5.5)	
Years of work practice			>0.05
≤ 2	152 (19.3)	21/152 (13.8)	
3–5	178 (22.6)	24/178 (13.5)	
6–10	250 (31.8)	23/250 (9.2)	
11–15	108 (13.7)	15/108 (13.9)	
≥16	91 (11.6)	8/91 (8.8)	
Healthcare Institution			
Primary Healthcare center	162 (20.6)	10/162 (6.2)	<0.05^**^
Secondary healthcare hospital	289 (36.8)	47/289 (16.3)	
Tertiary Healthcare hospital	335 (42.6)	34/335 (10.1)	
Area of practice			
Medicine/Medical department	239 (30.4)	23/239 (9.6)	<0.001^**^
Surgery/Surgical department	148 (18.8)	32/148 (21.6)	
Intensive Care Unit	66 (8.4)	6/66 (9.1)	
Emergency department	43 (5.5)	8/43 (18.6)	
OPD /PHC	94 (12.0)	3/94 (3.2)	
Obs-Gynae / Pediatrics	50 (6.4)	8/50 (16.0)	
Laboratory	23 (2.9)	2/23 (8.7)	
General practice	50 (6.4)	1/50 (2.0)	
Others	73 (9.3)	8/73 (11.0)	

* *p value- according to Chi-square test applied*.

** *Statistically significant*.

*p < 0.05 means statistically significant, p < 0.001 means highly statistically significant*.

**Figure 1:**
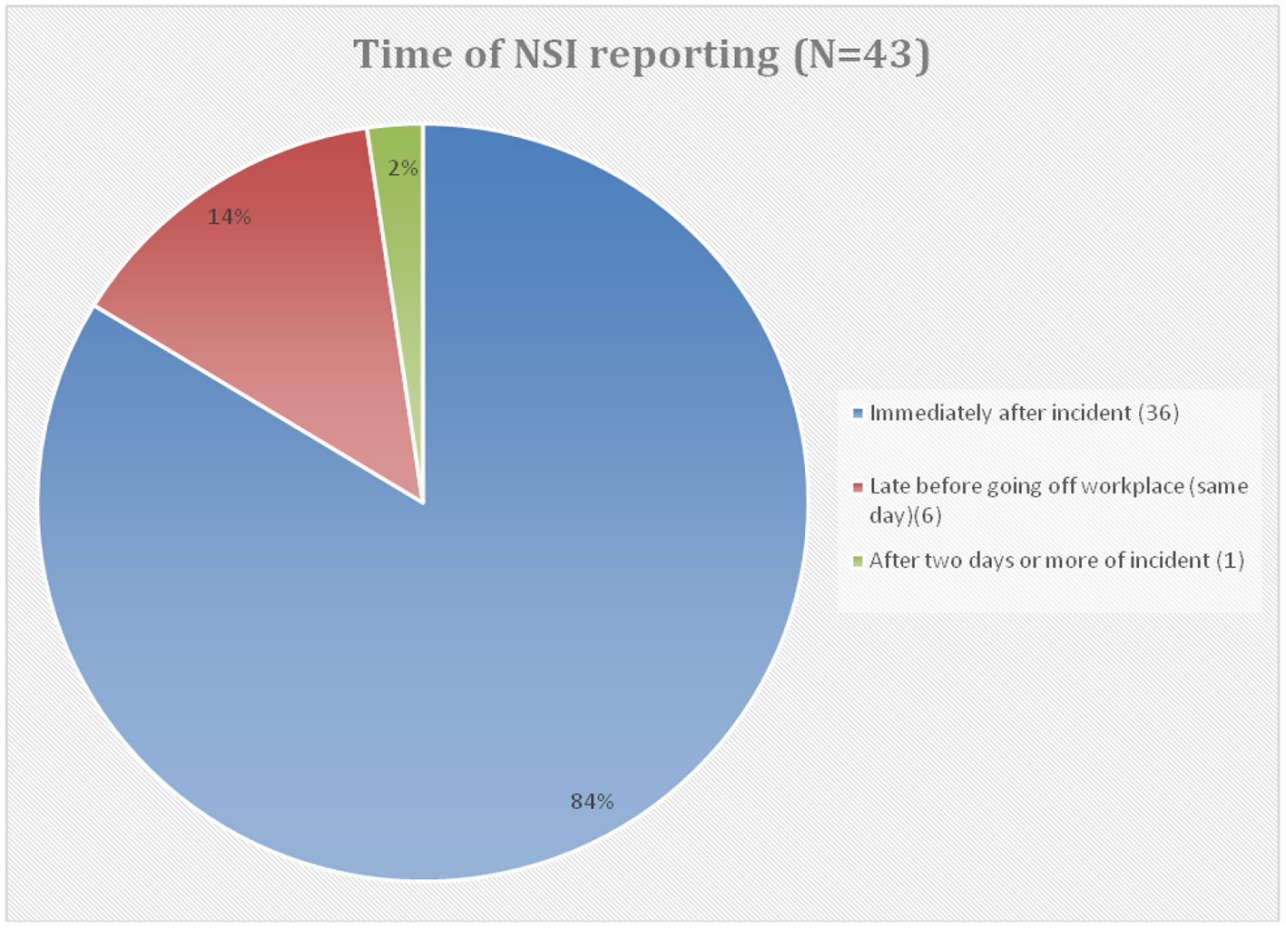
Time of NSI reporting.

**Figure 2:**
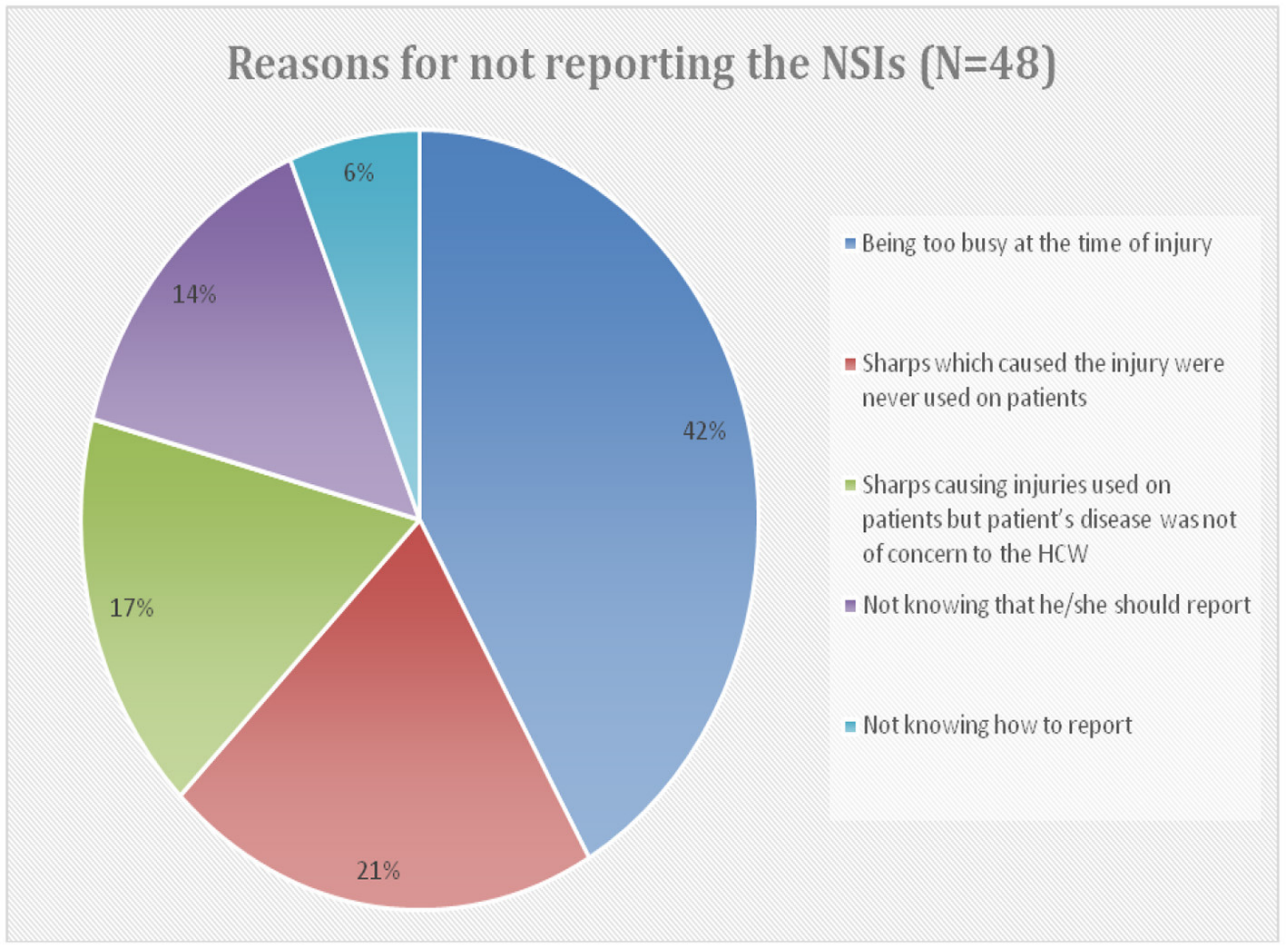
Reasons for not reporting the NSIs among those HCWs who did not report their NSIs (48/91).

The incidence of NSIs among HCWs in Abha city was 11.57%. Table 3 shows HCWs responses to items regarding NSIs in the previous year. Regarding the type of injury, 61.5% of respondents described their injuries as superficial (little or no bleeding), whereas 38.5% as moderate (skin punctured, some bleeding). Almost half (47.3%) of these injuries were reported by a HCW to appropriate authorities, and the majority of them (83.7%) reported immediately after the incident (Figure 1). However, 52.7% (48/91) did not report their injuries, and their reasons for not reporting were too busy at the time of injury (41.7%), did not know they should report (14.6%), and did not know how to report (6.3%). Nearly 20.8% stated that sharp devices caused injuries that were never used on a patient (Figure 2).

**Table 3:** Experiences regarding NSIs.

**Items**	**Frequency (%)**
**Number of NSI (*****N*** **=** **91)**	
Once	36 (39.6)
Two to four times	45 (49.5)
≥ five times	08 (8.8)
Don't remember	02 (2.1)
**Injury type (*****N*** **=** **91)**	
Superficial (little or no bleeding)	56 (61.5)
Moderate (skin punctured, some bleeding)	35 (38.5)
Severe (deep stick/cut, or profuse bleeding)	—-
**Reporting the NSI (*****N*** **=** **91)**	
Yes	43 (47.3)
No	48 (52.7)
**Receive medical attention within 2 h after injury (*****N*** **=** **91)**	
Yes	42 (46.2)
No	49 (53.8)
**Action taken after injury (Multiple responses question)**	
Washed with soap and water	70 (76.9)
Get tested for HIV, hepatitis B, and hepatitis C	40 (44.0)
Identify the source patient	35 (38.5)
Get post-exposure prophylaxis (PEP) when the source patient is unknown or tests positive for HIV, hepatitis B, and hepatitis C	21 (23.1)
**Device involved in the last incident (*****N*** **=** **91)**	
Intravenous (IV) cannula	30 (33.0)
Butterfly needle	5 (5.5)
Hypodermic needle	17 (18.7)
Phlebotomy needle	6 (6.6)
Lancets/ Razors/ Scissors	9 (9.9)
Suture needles	14 (15.4)
Others	10 (11.0)
**When the sharps injuries occurred (*****N*** **=** **91)**	
During use	48 (52.7)
After use and before disposal	20 (22.0)
Between steps in procedures	13 (14.3)
During disposal	5 (5.5)
While re-sheathing or recapping a needle	5 (5.5)
Work area where recent injury occurred (*N* = 91)	
Patient room	39 (42.9)
Outside patient room (hallway, nurses station, etc.)	4 (4.4)
Emergency department	12 (13.2)
Intensive/Critical care unit	5 (5.5)
Operating room/Recovery	24 (26.4)
Outpatient clinic/Office	5 (5.5)
Others	2(2.2)

In terms of the location of these injuries, 91.2% were in the hands, mainly fingers of which the right index finger represents the most common site (46.2%). About 76.9% of HCWs who sustained NSIs washed the injury site with soap and water, 38.5% identified the source patient, 44.0% got tested for HIV, hepatitis B, hepatitis C, and only 23.1% got post-exposure prophylaxis.

**Figure 3:**
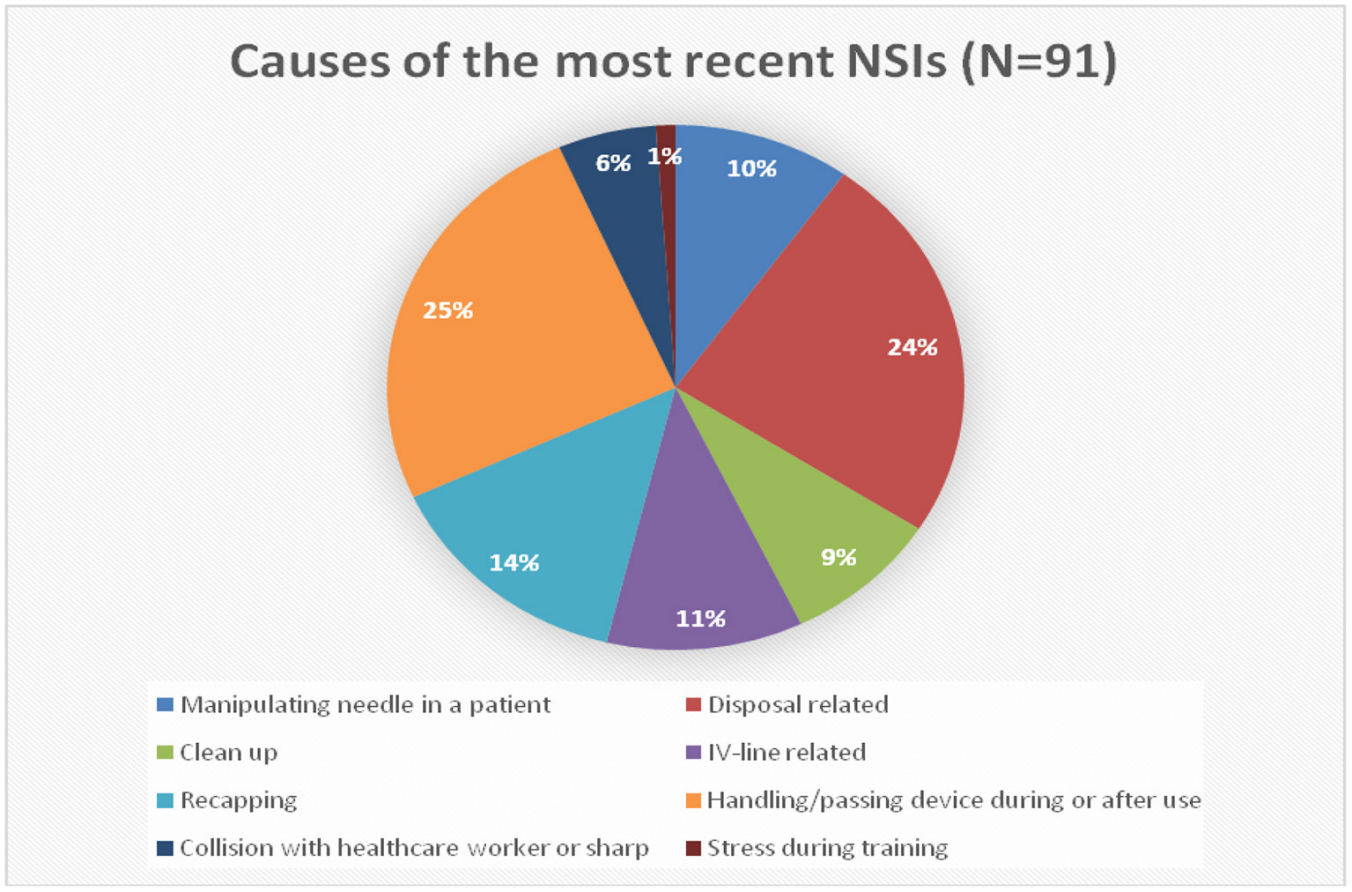
Causes of the most reasons NSIs.

An intravenous cannula (33.0%) followed by a hypodermic needle (18.7%) were the most common devices involved in most of NSIs. More than half of NSIs occurred during the use of sharp devices (52.7%), while 22.0% occurred after use and before disposal. About 42.9% of injuries happened in the patient room. From the HCW perspective, handling/passing devices during or after use (25.3%) and disposal-related causes (24.2%) were the significant causes of NSI, followed by recapping (14.3%). In comparison, stress training represents only 1.1% of all causes (Figure 3).

Factors such as level of healthcare and area of practice were found to be significantly associated with NSIs. The incidence of NSIs was significantly higher among those who worked in the secondary healthcare level (*p* = 0.003), and those who were practicing surgery (*p* < 0.001). Physicians, males, and younger HCWs reported more NSIs than others, but these differences were not statistically significant (Table 2).

Out of 786 respondents, 94.7% knew about the definition of NSI, and 82.4% of them were aware of sharps disposal containers recommendation, whereas 61.2% recognized the recap of the needle was not recommended. The majority of respondents (78.9%) gave a correct answer regarding the doses of the Hepatitis B vaccine, while 43.5% knew that there is no vaccine for Hepatitis C (Table 4).

**Table 4:** Percentages of correct responses to the knowledge questions related to NSI.

**Knowledge questions related to NSI**	**Percentage of correct responses**			
	**Total HCWs**	**HCWs injured with needle stick**	**HCWs not injured with needle stick**	*p* value^*^
	***N* = 786**	***N* = 91**	***N* = 695**	
**Prevention**				
NSI is defined as wounds caused by needles that accidentally puncture the skin. (Yes)	744 (94.7)	86 (94.5)	658 (94.7)	1.000
Recap of the needle after performing nursing procedures is recommended to decrease the risk of needlestick injury. (No)	481 (61.2)	51 (56.0)	430 (61.9)	0.283
Dispose in a sharps container after performing procedures is recommended to decrease the risk of needlestick injury. (Yes)	648 (82.4)	71 (78.0)	577 (83.0)	0.238
Three doses are required for full protection from Hepatitis B. (Yes)	620 (78.9)	72 (79.1)	548 (78.8)	0.952
Hepatitis C disease can be prevented by vaccine. (No)	342 (43.5)	37 (40.7)	305 (43.9)	0.559
**Disease transmission**				
Needle stick Injuries may transmit blood-borne diseases like hepatitis B virus (HBV), hepatitis C virus (HCV), and (HIV). (Yes)	727 (92.5)	85 (93.4)	642 (92.4)	0.725
Hepatitis B & C, HIV are blood-borne pathogens that Medical staff are most commonly exposed to when they experience needlestick injury. (Yes)	687 (87.4)	79 (86.8)	608 (87.5)	0.857
In needlestick injury, Hepatitis B carries the greatest risk of transmission. (Yes)	603 (76.7)	71 (78.0)	532 (76.5)	0.754
The percentage transmission of HBV is higher than HIV owing to needle stick injury. (Yes)	517 (65.8)	60 (65.9)	457 (65.8)	0.973
**Post exposure measures**				
Are you aware of the procedure and guidelines to follow if you sustain a needlestick injury in your workplace? **(Yes)**	637 (81.0)	70 (76.9)	567 (81.6)	0.286
If you have a needlestick injury your immediate action will be to wash your hand with water only. **(No)**	591 (75.2)	70 (76.9)	521 (75.0)	0.684
If you have a needlestick injury your immediate action will be to wash your hand with soap and water. **(Yes)**	592 (75.3)	72 (79.1)	520 (74.8)	0.371
If you have a needlestick injury your immediate action will be to wash your hand with antiseptic solution. **(No)**	391 (49.7)	44 (48.4)	347 (49.9)	0.777
There is currently no approved post-exposure prophylaxis for HCV. **(Yes)**	340 (43.3)	43 (47.3)	297 (42.7)	0.413
Concerning needle stick injury from HCV infected patient, HCV antibody testing should be performed at 4–6 months. **(Yes)**	460 (58.5)	57 (62.6)	403 (58.0)	0.397
Tetanus vaccine is part of the treatment after experiencing needlestick injury. (No)	180 (22.9)	23 (25.3)	157 (22.6)	0.567

* *p value- according to Chi-square test*.

Regarding the responses on diseases transmission questions, 92.5% of HCWs knew that NSIs could transmit HBV, HCV, and HIV, and 87.4% of them were aware that these are the most common diseases that medical staff is exposed to after NSI. Most of the respondents (76.7%) knew that Hepatitis B carries the most significant risk of transmission, while 65.8% knew that this risk is higher than HIV (Table 4).

When respondents were asked about the post-exposure measures, most (81.0%) were aware of the procedure and guidelines to follow if they sustained an NSI. About 58.5% of them correctly answered a question about HCV antibody testing, and 43.3% were aware that there is no approved post-exposure prophylaxis (PEP) for HCV. In comparison, only 22.9% knew that the tetanus vaccine is not a part of PEP (Table 4).

Regarding immediate action to be taken when exposed to NSI, 75.3, 75.2, and 49.7% of respondents gave correct responses to wash their hands with soap and water, water only, and antiseptic solution, respectively. There were no statistically significant differences in knowledge between HCWs who had and did not have NSIs (Table 4).

Two-thirds of the participants had a positive attitude toward worrying about having NSI. Most participants either strongly disagree (46.8%) or disagree (28.5%) that patient care is more important than their safety. The majority strongly agreed (59.4%) or agreed (30.9%) that all sharps injuries at work should be reported immediately. Almost half of the participants agreed, and 33.6% strongly agreed that NSI is preventable. Additionally, 93.5% had a positive attitude to the fact that a professional company should dispose of the needle and sharp objects waste (Table 5).

**Table 5:** Responses to the attitude statements.

**Items**	**Frequency (%)**				
	**Strongly disagree**	**Disagree**	**Neutral**	**Agree**	**Strongly agree**
I am worry about having needle stick injury.(+ve)	48 (6.1)	51 (6.5)	147 (18.7)	266 (33.8)	274 (34.9)
Patient care is more important than the safety of HCWs. (–ve)	368 (46.8)	224 (28.5)	108 (13.7)	37 (4.7)	49 (6.2)
All sharps injuries at work should be reported immediately. (+ve)	27 (3.4)	17 (2.2)	32 (4.1)	243 (30.9)	467 (59.4)
I think needle stick injury is preventable. (+ve)	24 (3.1)	46 (5.9)	82 (10.4)	370 (47.1)	264 (33.6)
Sharp objects waste should be disposed of by a professional company not in domestic waste. (+ve)	23 (2.9)	9 (1.1)	19 (2.4)	278 (35.4)	457 (58.1)

*+ve, positive statement; –ve, negative statement*.

Out of 786 respondents, 27.5% incorrectly practiced recapping the needles with two hands, and 8.7% bend needles before disposal. Regarding the disposal container, 95.3% confirm its availability, and 97.1% were always using it when disposed of sharp items. A majority of HCWs (89.1%) had been vaccinated against Hepatitis B, while only half of them had received training on the use of safety devices in the last year. Practicing recapping the needles with two hands before disposal was statistically significantly higher among HCWs who had a history of NSI (36.3 vs. 26.3%; *p* = 0. 046) (Table 6).

**Table 6:** Percentages of correct responses to the practice questions related to NSI.

**Practice questions related to NSI**	**Percentage of correct responses**			
	**Total HCWs**	**HCWs injured with needle stick**	**HCWs not injured with needle stick**	*p* value^*^
	***N* = 786**	***N* = 91**	***N* = 695**	
Do you recap needles with 2 hands before disposal? (NO)	570 (72.5)	58 (63.7)	512 (73.7)	0.046^**^
Do you bend needles before disposal? (NO)	718 (91.3)	84 (92.3)	634 (91.2)	0.729
Is the safety box/disposal container usually available? (YES)	749 (95.3)	84 (92.3)	665 (95.7)	0.182
Do you always put sharp items into its assigned disposal container? (YES)	763 (97.1)	88 (96.7)	675 (97.1)	0.742
Have you been vaccinated against Hepatitis B? (YES)	700 (89.1)	78 (85.7)	622 (89.5)	0.277
Have you received training on the use of safe devices in the last year? (YES)	415 (52.8)	48 (52.7)	367 (52.8)	0.992

* *p value- according to Chi-square test*.

** *Statistically significant*.

## Discussion

NSIs are one of the most important risks to HCWs during their careers. Several studies were conducted to determine the incidence rate of these injuries in KSA among HCWs related to the number of beds in their hospitals based on data records during different periods (3–9). Other studies have explored the incidence/prevalence of NSIs in specific populations like laboratory workers and dental assistants (22, 23).

In our study, the incidence of NSIs among HCWs was 11.57% during the previous 12 months. This finding was less than those (14% in Jazan and 15% in Abha respectively) reported in previous local studies conducted among primary HCWs (18, 19). Additionally, this incidence is also lower than the finding (19%) in UAE (27), 40 % in Iran (28), 22.7 % in Lebanon (29), and 67.9% in Egypt (30). Different studies have used different criteria to report the incidence, prevalence or needle stick injury rate making it difficult to compare them. The low incidence may be attributed to the regular training of HCWs in recent years in KSA (31). In addition, limiting the reported incidence to the previous 12 months and self-reporting of injuries in the questionnaire may underestimate the incidence. Information about the age and the tasks assigned to workers and the ratio of HCWs to the patients is important for a fair comparison. In this study, 42% of HCWs had less than five years of experience; there is no doubt that a large number of them are still working under supervision, and many of them are assigned to simple tasks.

As reported by different studies, most of NSIs were reported by Nurses (8, 9, 32), and the majority of injuries happened in the patient room (28, 32). In our study, intravenous (IV) cannula was the most common device involved in most incidents which are similar to what was reported by several studies. (3, 8, 9, 32).

Recapping the needles after use was reported as a common cause of NSIs in many studies (19, 33, 34). On the contrary, in our study, the handling/passing device during or after use and disposal-related causes (24.2%) were the significant causes, while recapping the needle accounted for only 14% of all incidents.

Underreporting of sharp injuries is a common problem in healthcare facilities worldwide (11, 35). In this study, almost half (47.3%) of these injuries were reported by HCWs to appropriate authorities. This is consistent with that reported from Poland (55%) (36) and UK (51%) (37), but it is lower than that (80%) reported from UAE (38) and India (32). Nearly 6.0% of respondents did not know how to report a NSI in the present study which is comparable to the UK study [8%], whereas 14% HCWs in our study were not aware that they should report a NSI which is again comparable to findings of the UK study (37).

According to post-exposure actions, 76.9% of HCWs who sustained NSIs in this study washed the injury site with soap and water compared to 66% in India (33) and only 22% in Nepal (39). Additionally, we observed only in 38.5% of all incidents the source patients were identified, which was lower than that reported by the local studies, i.e. 73% in both Al Ahsa region (10) and University Hospital in Al Riyadh (6) and 84.4% in Najran (8). However, this difference may be explained by the reason that these above local studies were based on data obtained from hospital records.

The incidence of NSIs was significantly higher among those practicing surgery as their specialty. This finding is consistent with other studies (5, 27, 40, 41). Also, we found a significantly higher incidence of NSIs among HCWs who worked in secondary healthcare hospitals than tertiary hospitals (16.3 vs. 10.1%). Similarly, the needle stick and sharps injuries rates were 30 and 14% in secondary and tertiary hospitals, respectively in a study conducted in Jazan (42). The difference in the health services, numbers, and types of procedures in addition to the number of admissions may explain the difference in incidence between secondary and tertiary healthcare hospitals in this study. More studies are needed to explore these differences.

Additionally, physicians, males, and younger HCWs reported more NSIs than others, but these differences were not statistically significant, which coincides with the results of a similar Iranian study (28). However, on the contrary, a study from China shows a significant association between NSIs with gender, age, and job position (43).

In this study, 43.5% knew there is no vaccine for Hepatitis C which is in contrast with the finding (75%) observed by Jankovic et al. (44).

In the current study, 92.5% of HCWs knew that HBV, HCV, and HIV could be transmitted by NSIs, which is consistent with the findings of a Malaysian study (43) but higher than those reported in Bosnia (45) and Delhi (33). In this study, only 65.8% knew that this risk of Hepatitis B transmission is higher than the risk of HIV, which is less than that observed (82%) in the Irish study (46).

Moreover, in the present study, 82.4% were aware of sharps disposal containers recommendation, which is better than that reported (29%) by a study from the USA (47). Most HCWs in this study (81.0%) were aware of the PEP and Universal precaution guidelines, which is better than that (61%) reported in a local study from Sarourah (21) but it is lower than the observations seen in Indian (48) and Malaysian (44) studies. Only 43.3% of HCWs in this study were aware that there is no PEP for HCV and 58.5% knew the timing of HCV antibody testing. This low knowledge regarding HCV post prophylaxis is also seen in other studies (32, 49).

In a recent study conducted among dental assistants in Jeddah, it was found that disease transmission decreased the risk of NSIs, and this association was statistically significant (22). However, in this study, we find there are no statistically significant differences in knowledge between HCWs who had and who did not have NSI. Our finding is consistent with that reported by Abuduxike et al. in the Cyprus study (50).

Our study shows only two-thirds of the participants had a positive attitude toward worrying about having NSI at work. This is lower than that reported by a Sudan study where 83% of HCWs were worried about these injuries (32). Similar to a local study among HCWs who work in primary health centers in the Jazan region (19), this study finds most HCWs agree that the needle and sharp objects waste should be disposed of by a professional company.

In our study, the majority show a negative attitude toward patient care is important than HCWs safety which is consistent with the attitude of Sudanese HCWs (31). A study conducted in China (41) had reported that HCW's behaviors and attitudes were significantly related to NSIs at work, whereas the Cyprus Study (50) found no significant relationship between the attitude of HCWs and the experience of NSI. Although there are recommendations against recapping the needles after use (51), this practice is still prevalent among HCWs. Several studies from different countries have reported that as the leading cause of NSIs. This risky practice was reported by 66.3% HCWs in India (33) and 46% in Cyprus (50). However, only 5.8% in Malaysia (45) and 13.4% in Lebanon (29) reported this practice. In our study, 27.5% of participants incorrectly practiced recapping the needles with two hands.

Availability of disposal container is an important matter, 95.3% of HCWs in this study confirm its availability, and almost all of them always use it when disposing of sharp items, which is comparable to other studies (18, 28, 50).

A majority of our study sample (89.1%) had been vaccinated against Hepatitis B, which is consistent with reports from local studies (18)(19). Studies have reported a high rate of Hepatitis B vaccination among HCWs, i.e. 100 % in Iran (28), 91.5% in India (33), 88.4% in Lebanon (29), 77% in Bahrain (52) and ranged between 62 and 80% in the United Arab Emirates (27, 38). This high percentage in our study could be due to the fact that the vaccination is free of charge and the pre-employment checkup exists for all HCWs in KSA.

Our study has a few limitations. The cross-sectional design cannot confirm the causality of the relationship between compared variables. The self-reported response could over or underestimate the result. The study's weakness is that it was conducted in a single city of the Aseer Region of KSA. We hope in the future to have all the required resources to do multicentric /nationwide studies. However, a representative sample including HCWs from all levels of health care is the strength of our study.

## Conclusions

The exposure of healthcare professionals to needle stick injury and its underreporting is still a prevalent issue. In this study, during the past 12 months, the incidence of needle stick injury among healthcare workers was 11.57% and more than half of the injuries went unreported. Future studies need to explore the risk factors of NSIs and to assess the benefit of the preventive measures on reducing the risk. Increasing awareness among HCWs and providing regular training on the safe use of sharp devices is highly recommended. Improving the current reporting systems for NSIs to ensure early use of post-exposure prophylaxis is also recommended. Implementation of safety precautions and safe injection practices and providing engineered safety devices may further help in reducing the risk of NSIs.

## Data Availability Statement

The raw data supporting the conclusions of this article will be made available by the authors, without undue reservation.

## Author Contributions

AA conceived the idea of this study, supervised the study, participated in the design of the research instrument, reviewed related literature, and participated in discussing findings and making recommendations on the basis of the findings of the study. NA conceived the idea of this study, participated in the design of the study, and had the major responsibility of coordinating the data collection. SA and JA-L participated in design of the work, interpretation of data, and writing of the manuscript. MA and SS participated in data collection, study subjects management, and manuscript writing. AAA participated in design of the work, analysis of the data, and interpretation of the results. SM and MA finalized the manuscript for submission. All authors have read and approved the final manuscript.

## Conflict of Interest

The authors declare that the research was conducted in the absence of any commercial or financial relationships that could be construed as a potential conflict of interest.

## Publisher's Note

All claims expressed in this article are solely those of the authors and do not necessarily represent those of their affiliated organizations, or those of the publisher, the editors and the reviewers. Any product that may be evaluated in this article, or claim that may be made by its manufacturer, is not guaranteed or endorsed by the publisher.

## References

[1] World Health Organization. The World Health Report. Geneva: WHO. Available online at: http://www.who.int/whr/2002/chapter4/en/index8.html.g (accessed January 15, 2022).

[2] Centers for Disease Control and Prevention: NIOSH Publications and Products. Preventing Needlestick Injuries in Health Care Settings (2000-108). Available online at: http://www.cdc.gov/niosh/docs/2000-108/ (accessed June 11, 2015).

[3] Memish ZA Assiri AM Eldalatony MM Hathout HM. Benchmarking of percutaneous injuries at the Ministry of Health hospitals of Saudi Arabia in comparison with the United States hospitals participating in Exposure Prevention Information Network (EPINet™). Int J Occup Environ Med. (2015) 6:26–33. 10.15171/ijoem.2015.467 25588223 PMC6977063

[4] Memish ZA Assiri AM Eldalatony MM Hathout HM Alzoman H Undaya M. Risk analysis of needle stick and sharp object injuries among health care workers in a tertiary care hospital (Saudi Arabia). J Epidemiol Glob Health. (2013) 3:123–9. 10.1016/j.jegh.2013.03.004 23932054 PMC7320367

[5] Samargandy SA Bukhari LM Samargandy SA Bahlas RS Aldigs EK Alawi MM. Epidemiology and clinical consequences of occupational exposure to blood and other body fluids in a university hospital in Saudi Arabia. Saudi Med J. (2016) 37:783–90. 10.15537/smj.2016.7.14261 27381540 PMC5018644

[6] El-Hazmi Malak M Al-Majid Fahad M. Needle stick and sharps injuries among health care workers: a 5-year surveillance in a teaching center in Saudi Arabia. Biomed Res. (2008) 19:133–40.

[7] Jahan S. Epidemiology of needlestick injuries among health care workers in a secondary care hospital in Saudi Arabia. Ann Saudi Med. (2005) 25:233–8. 10.5144/0256-4947.2005.233 16119525 PMC6147994

[8] Hashmi A Al Reesh SA Indah L. Prevalence of needle-stick and sharps injuries among healthcare workers, Najran, Saudi Arabia. Epidemiology. (2012) 2:117. 10.4172/2161-1165.1000117

[9] Elsherbenny E Niazy NA. Incidence of Needle Stick and Sharps Injuries Among Health Care Workers in a Tertiary Hospital, KSA Egypt. J Occup Med. (2018) 42:271–84. 10.21608/ejom.2018.6810

[10] Al Shaikh H Al Mahdi M Naik B. Sharps injuries among health care workers in Al Ahsa region, Saudi Arabia. Int J Infect Control. (2019) 15:1–8. 10.3396/ijic.v15i4.017.19

[11] Elder A Paterson C. Sharps injuries in UK health care: a review of injury rates, viral transmission and potential efficacy of safety devices. Occup Med (Lond). (2006) 56:56674. 10.1093/occmed/kql122 17065314

[12] Prüss-Üstün A Rapiti E Hutin YJF. (2003). Sharps Injuries: Global Burden of Disease From Sharps Injuries to Health-Care Workers. Available online at: https://apps.who.int/iris/handle/10665/42743 (accessed January 15, 2022).

[13] Wilburn SQ Eijkemans G. Preventing needlestick injuries among healthcare workers: a WHO-ICN collaboration. Int J Occup Environ Health. (2004) 10:451–6. 10.1179/oeh.2004.10.4.451 15702761

[14] Gupta A Anand S Sastry J Krisagar A Basavaraj A Bhat SM. High risk for occupational exposure to HIV and utilization of post-exposure prophylaxis in a teaching hospital in Pune, India. BMC Infect Dis. (2008) 8:142. 10.1186/1471-2334-8-142 18939992 PMC2588594

[15] Sohn JW Kim BG Kim SH Han C. Mental health of healthcare workers who experience needlestick and sharps injuries. J Occup Health. (2006) 48:474–9. 10.1539/joh.48.474 17179640

[16] Tarigan L Cifuentes M Quinn M Kriebel D. Prevention of needle-stick injuries in healthcare facilities: a meta-analysis. Infect Control Hosp Epidemiol. (2015) 36:823–9. 10.1017/ice.2015.50 25765502

[17] Benn P Fisher M Kulasegaram R. UK guideline for the use of post-exposure prophylaxis for HIV following sexual exposure (2011). Int J Std AIDS. (2011) 22:695–708. 10.1258/ijsa.2011.171011 22174049

[18] Mahfouz AA Abdelmoneim I Khan MY Daffalla AA Diab MM Shaban H. Injection safety at primary health care level in south-western Saudi Arabia. East Mediterr Health J. (2009) 15:443–50. 10.26719/2009.15.2.443 19554992

[19] Ismail AA Mahfouz MS Makeen A. Injection safety among primary health care workers in Jazan Region, Saudi Arabia. Int J Occup Environ Med. (2014) 5:155–63. 25027044 PMC7767601

[20] Alzahrani W Almatrafi Y Alzahrani Y Alahmadi Y Allehyani E Alahmadi A. Frequency of needlestick injury with associated risk factors and knowledge about blood borne transmitted diseases among health care workers in Alnoor Specialist Hospital, Makkah, Saudi Arabia. Int J Sci Res. (2017) 6:830–3. 10.21275/28121601

[21] Alam M. Knowledge, attitude and practices among health care workers on needle-stick injuries. Ann Saudi Med. (2002) 22:396–9. 10.5144/0256-4947.2002.396 17146275

[22] AlDakhil L Yenugadhati N Al-Seraihi O Al-Zoughool M. Prevalence and associated factors for needlestick and sharp injuries (NSIs) among dental assistants in Jeddah, Saudi Arabia. Environ Health Prev Med. (2019) 24:60. 10.1186/s12199-019-0815-7 31601166 PMC6788026

[23] Khabour OF Al Ali KH Mahallawi WH. Occupational infection and needle stick injury among clinical laboratory workers in Al-Madinah city, Saudi Arabia. J Occup Med Toxicol 13, 15. (2018). 10.1186/s12995-018-0198-5 29942343 PMC5963129

[24] Lwanga SK Lemenshow S. Sample Size Determination in Health Studies. A Practical Manual. Geneva: World Health Organization (1991).

[25] Ministry of Health. Employee Health Program Policy. (2018). p. 0–55. Available online at: http://gdipc.org/wp-content/uploads/2018/07/Employee-Health-Program-Policy-2018.pdf (accessed January 15, 2022).

[26] “GCC Infection control manual 2013 revised OPT,” 2013, [Online]. Available: https://www.moh.gov.sa/CCC/Documents/GCC Infection control manual 2013 revisedOPT.pdf. (accessed January 15, 2022).

[27] Jacob A Newson-Smith M Murphy E Steiner M Dick F. Sharps injuries among health care workers in the United Arab Emirates. Occup Med (Lond). (2010) 60:395–7. 10.1093/occmed/kqq039 20407045

[28] Yarahmadi R. The prevalence of needle sticks injuries among health care workers at a hospital in Tehran. Iran J Health Saf Environ. (2014) 1:23–9.

[29] Sabbah I Sabbah H Sabbah S Akoum H Droubi N. Occupational exposures to blood and body fluids (BBF): Assessment of knowledge, attitude and practice among health care workers in general hospitals in Lebanon. Health. (2013) 5:70–8.

[30] Hanafi MI Mohamed AM Kassem MS Shawki M. Needlestick injuries among health care workers of University of Alexandria Hospitals. East Mediterr Health J. (2011) 17:26–35. 10.26719/2011.17.1.26 21735798

[31] Bouya S Balouchi A Rafiemanesh H Amirshahi M Dastres M Moghadam MP. Global prevalence and device related causes of needle stick injuries among health care workers: a systematic review and meta-analysis. Ann. Global Health. (2020) 86:35 10.5334/aogh.2698 32346521 PMC7181946

[32] Dafaalla MD Suliman A. Knowledge, attitude and practice towards needle stick injury among health care workers in a tertiary Sudanese Hospital. S Am J Clin Res. (2016) 3:88–96. 10.21522/TIJCR.2014.03.01.Art010

[33] Sharma S Gupta A Arora A. Knowledge, attitude and practices on needle-stick and sharps injuries in tertiary care cardiac hospital: a survey. Indian J Med Sci. (2010) 64:396–401. 10.4103/0019-5359.101174 23006418

[34] Lal D Sidhu TK Coonar PS Singh G. Needle stick injuries among health care workers in a tertiary care hospital in District Bathinda, Punjab. Indian J Commun Health. (2017) 29:429–33.

[35] Kim OS Jeong JS Kim KM Choi JS Jeong IS Park ES. Underreporting rate and related factors after needlestick injuries among healthcare workers in small- or medium-sized hospitals. Korean J Nosocomial Infect Control. (2011) 16:29–36.

[36] Garus-Pakowska A Górajski M. Behaviors and attitudes of polish health care workers with respect to the hazards from blood-borne pathogens: a questionnaire-based study. Int J Environ Res Public Health. (2019) 16:891. 10.3390/ijerph16050891 30870976 PMC6427109

[37] Elmiyeh B Whitaker IS James MJ Chahal CA Galea A Alshafi K. Needle-stick injuries in the National Health Service: a culture of silence. J R Soc Med. (2004) 97:326–7. 10.1177/014107680409700705 15229257 PMC1079524

[38] Zaidi MA Griffiths R Beshyah SA Myers J Zaidi MA. Blood and body fluid exposure related knowledge, attitude and practices of hospital based health care providers in United Arab Emirates. Saf Health Work. (2012) 3:209–15. 10.5491/SHAW.2012.3.3.209 23019533 PMC3443696

[39] Singh B Paudel B Kc S. Knowledge and practice of health care workers regarding needle stick injuries in a tertiary care center of Nepal. Kathmandu Univ Med J. (2015) 51:230–3. 10.3126/kumj.v13i3.16813 27180369

[40] Rajkumari N Thanbuana BT John NV Gunjiyal J Mathur P Misra MC. Prospective look at the burden of sharps injuries and splashes among trauma health care workers in developing countries: true picture or tip of iceberg. Injury. (2014) 45:1470–8. 10.1016/j.injury.2014.03.001 24680470

[41] Afridi AAK Kumar A Sayani R. Needle stick injuries– risk and preventive factors: a study among health care workers in tertiary care hospitals in Pakistan. Glob J Health Sci. (2013) 5:85–92. 10.5539/gjhs.v5n4p85 23777725 PMC4776811

[42] Makeen AM Alharbi AA Mahfouz MS Alqassim AY Ismail AA Arishi HM. Needlestick and sharps injuries among secondary and tertiary healthcare workers, Saudi Arabia. Nurs Open. (2021) 9:816–23. 10.1002/nop2.1136 34806326 PMC8685775

[43] Wang C Huang L Li J Dai J. Relationship between psychosocial working conditions, stress perception, and needle-stick injury among healthcare workers in Shanghai. BMC Public Health. (2019) 19:874. 10.1186/s12889-019-7181-7 31272426 PMC6610837

[44] Jankovic S Bojanic J Jovic-Vranes A Marinkovic J Jankovic J. Knowledge, attitudes and practices towards blood-borne pathogens in healthcare workers in Banja Luka, Bosnia and Herzegovina. Cent Eur J Med. (2009) 4:409–14. 10.2478/s11536-009-0087-5

[45] Rampal L Zakaria R Sook LW Zain AM. Needle stick and sharps injuries and factors associated among health care workers in a Malaysian hospital. Eur J Soc Sci. (2010) 13:354–62.

[46] Ö'Connor MB Hannon MJ Cagney D Harrington U O'Brien F Hardiman N. A study of needle stick injuries among non-consultant hospital doctors in Ireland. Irish J Med Sci. (2011) 180:445–9. 10.1007/s11845-010-0667-z 21188543

[47] Butsashvili M Kamkamidze G Kajaia M Morse DL Triner W Dehovitz J. Occupational exposure to body fluids among health care workers in Georgia. Occup Med (Lond). (2012) 62:620–6. 10.1093/occmed/kqs121 22869786 PMC3612004

[48] Singh S Singh B Singh S Khurana A Verma R. Study of knowledge, attitude and practice among nurses regarding needle stick and sharp item injuries. Int J Community Med Public Health. (2019) 6:2064–8. 10.18203/2394-6040.ijcmph20191819

[49] Hebo HJ Gemeda DH Abdusemed KA. Hepatitis B and C Viral Infection: Prevalence, Knowledge, Attitude, Practice, and Occupational Exposure among Healthcare Workers of Jimma University Medical Center, Southwest Ethiopia. Sci World J. (2019) 2019:9482607. 10.1155/2019/9482607 30853866 PMC6377947

[50] Abuduxike G AcarVaizoglu S Asut O Cali S. An assessment of the knowledge, attitude, and practice toward standard precautions among health workers from a hospital in Northern Cyprus. Saf Health Work. (2021) 12:66–73. 10.1016/j.shaw.2020.09.003 33732531 PMC7940130

[51] Becker MH. Department of Health Behavior and Health Education, School of Public Health. Ann Arbor, ML: The University of Michigan.

[52] Matlab M Cowman S Al-Shagag A Aboabdo M. Needle stick injuries and compliance among doctors and nurses, Bahrain. Med Bull. (2017) 39:225–8. 10.12816/0047772

